# Efficacy of 5-Aminolevulinic Acid Photodynamic Therapy Versus Observation for Cervical Low-Grade Squamous Intraepithelial Lesions with High-Risk HPV Infection

**DOI:** 10.3390/pharmaceutics18101216

**Published:** 2026-09-24

**Authors:** Xiaoyun Wang, Jianxia Huang, Qiong He, Jianwei Zhou

**Affiliations:** Department of Gynecology, The Second Affiliated Hospital of Zhejiang University School of Medicine, Hangzhou 310009, China

**Keywords:** photodynamic therapy, efficacy, HPV, cervical low-grade squamous intraepithelial lesion, observation

## Abstract

**Objectives:** This study aimed to evaluate the efficacy of photodynamic therapy (PDT) compared to observation in managing low-grade squamous intraepithelial lesions (LSILs) with high-risk HPV infection. **Methods:** We conducted a retrospective cohort study involving 608 patients with pathologically confirmed LSILs and Hr-HPV infection, divided into PDT or observation groups based on clinical preference and evaluation. PDT was administered as a routine clinical treatment option at our center. Follow-up at 6 months included HPV testing, cytology, and colposcopy. **Results:** The PDT group exhibited significantly higher regression rates (92.5% vs. 48.3%) and complete HPV clearance rates (80.8% vs. 37.3%) than the observation group. Adverse effects from PDT were mild and transient, with no cervical scarring or skin photosensitivity observed. Ten patients conceived successfully post-PDT, with five uncomplicated deliveries to date. **Conclusions:** These findings suggest that PDT is an effective, non-invasive treatment for LSILs with Hr-HPV infection, demonstrating favorable regression and safety profiles while preserving cervical integrity, particularly in reproductive-aged women.

## 1. Introduction

Persistent infection with high-risk human papillomavirus (Hr-HPV) is a major cause of cervical intraepithelial neoplasia (CIN) and cervical cancer [1]. According to the 2014 WHO classification of female reproductive diseases, CIN I and certain cases of CIN II (P16-negative) are categorized as low-grade squamous intraepithelial lesions (LSILs). Studies indicate that approximately 60% of LSIL cases in the cervix spontaneously regress, 30% persist, and around 10% progress to high-grade squamous intraepithelial lesions (HSILs) within two years [2]. In principle, follow-up observation is recommended for LSIL management. However, in clinical practice, due to the nearly 40% risk of persistence or progression of LSIL, many patients opt against observation due to psychological stress and request active treatment [3]. Conventional treatments for LSIL include cervical electrocautery, laser therapy, loop electrosurgical excision procedure (LEEP), and conization. These interventions may result in significant side effects, such as prolonged postoperative bleeding, structural damage to cervical tissue, scarring, stenosis, miscarriage, or preterm birth [4]. Therefore, safer and less invasive alternatives are urgently needed.

Photodynamic therapy (PDT) is a highly selective, minimally invasive therapeutic modality that combines photosensitizing agents with light irradiation. 5-Aminolevulinic acid (5-ALA) is a naturally occurring precursor in the heme biosynthesis pathway. When administered exogenously, 5-ALA is converted intracellularly to the potent photosensitizer protoporphyrin IX (PpIX) through the heme biosynthetic pathway. Because rapidly dividing dysplastic cells preferentially accumulate PpIX, 5-ALA-PDT achieves selective destruction of abnormal cervical tissue while sparing healthy epithelium [5]. Kennedy et al. first demonstrated the clinical potential of ALA-based PDT and photodiagnosis nearly three decades ago [6]. Since then, 5-ALA-PDT has been successfully employed for cervical condyloma, CIN, and other female reproductive tract disorders [7]. The favorable safety profile of 5-ALA—including rapid systemic clearance of PpIX within 24 h and minimal skin photosensitivity—makes it particularly attractive for cervical lesions in reproductive-aged women [8]. Beyond direct cytotoxicity, 5-ALA-PDT modulates the local cervical microenvironment by activating immune cells and inflammatory cytokines and restoring lactobacillus abundance, thereby facilitating HPV clearance [9].

Despite the established efficacy of 5-ALA-PDT for cervical lesions, several important questions remain unanswered. First, most prior studies have focused on CIN rather than LSILs specifically, and the optimal treatment protocols for LSIL with persistent Hr-HPV infection have not been standardized. Second, the relative benefit of PDT versus observation for this specific population—particularly in terms of HPV clearance and cervical structural preservation—has not been comprehensively evaluated in large cohorts. Third, the clinical and virological factors that predict treatment response remain poorly defined. The present study addresses these gaps by retrospectively analyzing 608 patients with histologically confirmed LSILs and persistent Hr-HPV infection, comparing 5-ALA-PDT with observation. Our study specifically evaluates lesion regression, HPV clearance, safety, and cervical integrity, with the goal of providing evidence to guide individualized clinical decision-making for this increasingly diagnosed population.

## 2. Materials and Methods

### 2.1. Study Design and Data Collection

This was a retrospective cohort study. A total of 608 patients with histologically confirmed low-grade squamous intraepithelial lesions (LSILs) of the cervix who underwent colposcopy in the outpatient department between April 2019 and November 2024 were reviewed. All cervical biopsy specimens were independently reviewed and confirmed by two experienced gynecologic pathologists. All patients were diagnosed with persistent Hr-HPV infection, defined as continuous positivity for the same HPV genotype at intervals of ≥6 months for more than 12 months, as confirmed by HPV-DNA testing. PDT has been used as a routine clinical treatment option for cervical precancerous lesions and persistent HR-HPV infection at our center since 2019. Treatment allocation was non-randomized and based on patient preference and gynecologists’ recommendations, reflecting routine clinical practice rather than a prospective research protocol. Written informed consent was obtained from all participants for the use of their clinical data and follow-up information. This study was approved by the Institutional Ethics Committee of the Second Affiliated Hospital of Zhejiang University School of Medicine (protocol code IH2021066, approved on 30 November 2021).

Based on patient preference and gynecologists’ recommendations, the 608 patients were allocated to either the PDT group (N = 308) or the observation group (N = 300). The exclusion criteria were as follows: (1) unsatisfactory colposcopic findings or lesions extending into the endocervical canal; (2) history of physical therapy or surgical treatment for cervical disease within the past year; (3) receipt of adjuvant or alternative treatments following PDT or cold knife conization (CKC); (4) presence of severe comorbidities requiring concurrent treatment (e.g., cardiovascular disease, arrhythmia, or active systemic infection); (5) pregnancy; (6) hypersensitivity to porphyrins or 5-ALA.

### 2.2. 5-ALA PDT

A 19% 5-ALA solution was prepared by mixing 354 mg of ALA powder (Shanghai Fudan-Zhangjiang BioPharmaceutical Co., Ltd., Shanghai, China) with 1.5 mL of thermogel. The thermogel was a poloxamer 407-based thermosensitive hydrogel (Baiyuefu^®^ Moisturizing Gel, Cosmetology Solutions; Shanghai Taoran Biotechnology Co., Ltd., Shanghai, China; composition: water, poloxamer 407, oxides, 1,3-propanediol, and 1,2-hexanediol). A slender cotton swab soaked with the photosensitizer was inserted into the cervical canal. A sterile cotton pad (4 × 3 cm) was then applied to the ectocervix according to the lesion location. A gauze-filled condom was placed intravaginally to secure the cotton materials and maintain stable contact with the cervical tissue. During the 4-h incubation period, patients were asked to remain seated or in a semi-reclining position in an outpatient chair, to avoid strenuous physical activity, and to report any discomfort. After incubation, the cervical surface was irradiated with a 635 nm LED light source (LED-635, Wuhan Yage Photo-Electronic Co., Ltd., Wuhan, China) at a dose of 100 J/cm^2^. For the cervical canal, a cylindrical diffuser (length 26 cm, diameter 3.3 mm) coupled with a 635 nm diode laser (LD600-C; Wuhan Yage Photo-Electronic Co., Ltd., Wuhan, China) was used to deliver the same irradiance over 30 min. A total of 3 treatment sessions were administered at intervals of 7–14 days. Patient discomfort and side effects were recorded during the treatment period.

### 2.3. Follow-Up and Outcome Assessment

All patients underwent follow-up evaluations at 6 months post-treatment, including HPV-DNA testing, cervical cytology, and colposcopy-directed biopsy. In this study, HPV-DNA detection was performed using a hybridization probe assay platform, which simultaneously detects 24 high-risk HPV types, including 16, 18, and 22 other types (6, 11, 42, 43, 44, 81, 83, 31, 33, 35, 39, 45, 51, 52, 53, 56, 58, 59, 66, 68, 73, 82). Given the established highest oncogenic risk of HPV 16 and 18, these two types were analyzed individually. The remaining 22 high-risk types were grouped as “other HR-HPV” because each individual type was detected in too few patients to permit meaningful stratified analysis; this grouping approach is consistent with common practice in the HPV literature. Cytology was assessed via ThinPrep cytology tests (TCT), and cytological findings were interpreted strictly in accordance with the 2014 Bethesda System for Reporting Cervical Cytology, with diagnostic categories including negative for intraepithelial lesions or malignancy (NILM), atypical squamous cells of undetermined significance (ASCUS), LSILs, and high-grade squamous intraepithelial lesions (HSILs). Histological evaluations adhered to the 2014 World Health Organization Classification.

Cure was defined as negative HPV-DNA results, NILM on TCT, and an absence of LSILs on colposcopy-directed biopsy. Persistence was defined as pathological confirmation of an LSIL on cervical biopsy, and disease progression was defined as pathological progression to HSILs. Complete HPV clearance was defined as negative results for all detected HPV subtypes. Partial HPV clearance was defined as the clearance of partial infecting HPV genotypes while one or more high-risk HPV subtypes remained persistently positive. Both persistent positivity for the original HPV subtypes and partial HPV clearance were categorized as overall HPV-positive status. Colposcopy-directed cervical biopsy was performed for patients with persistent HPV positivity at the 6-month follow-up. All pathological results were independently reviewed and jointly confirmed by two experienced gynecologic pathologists.

### 2.4. Statistical Analysis

All data were analyzed using IBM SPSS Statistics Version 24.0. Results were expressed as counts and percentages (N [%]), and chi-square tests were employed for group comparisons. Univariate logistic regression analysis was performed to identify clinical factors associated with lesion remission and HPV clearance. Variables with *p* < 0.05 in the univariate analysis were included in a multivariate logistic regression model. Odds ratios (ORs) and 95% confidence intervals (CIs) were calculated. Differences were considered statistically significant at *p* < 0.05.

## 3. Results

### 3.1. Study Cohort Characteristics

A total of 608 female patients were included in the study. Of these, 308 were assigned to the PDT group and 300 to the observation group. The mean age of the PDT group was 35.6 years (range: 20–68 years), while that of the observation group was 36.5 years (range: 21–62 years). No significant differences were observed between the two groups with respect to age, HPV infection status, TCT results, or cervical transformation zone characteristics (Table 1).

### 3.2. Comparison of Lesion Regression Rate

After 6 months of follow-up, the lesion regression rate in the PDT group was 92.5%, compared to 48.3% in the observation group. Statistical analysis revealed that the lesion regression rate in the PDT group was significantly higher than that in the observation group (*p* < 0.01). Among patients whose lesions did not regress, two patients in the PDT group progressed to HSILs/CINII within 6 months: one 45-year-old patient with HPV 16/51/61 positive and multiple point LSILs; another 34-year-old patient with HPV 16 positive and also multiple point LSILs. Both patients ultimately underwent partial cervical resection. In the observation group, a total of 35 patients progressed to HSILs and subsequently received surgical treatment (Table 2). No significant statistical differences were observed in the effects of factors such as age, HPV infection type, or cytological findings on the lesion regression rate between the two groups.

### 3.3. Comparison of HPV Clearance Rate

As illustrated in Figure 1, the photodynamic therapy group demonstrated a significantly higher efficacy rate and an HPV clearance rate exceeding that of the observation group by more than 40%. Specifically, among the 308 patients in the PDT group, 249 (80.8%) achieved complete HPV negativity. Of the remaining patients, 20 (6.5%) exhibited partial clearance of multiple HPV infections, while 39 (12.7%) showed persistent HPV infection. In contrast, among the 300 patients in the observation group, only 112 (37.3%) became HPV negative; 56 (18.7%) experienced partial clearance, and 132 (44.0%) maintained persistent HPV infection. Statistical analysis confirmed a significant difference in HPV clearance rate between the two groups (*p* < 0.01) (Table 3).

### 3.4. Cost Outlay and Adverse Reactions

PDT involves 3 treatment sessions, each requiring a half-day visit to the hospital. During the 30-min light exposure, patients are required to remain supine; however, phone use is permitted. Outside of the light exposure period, patients may move about freely. The total cost for the three sessions is approximately 9000 yuan, which must be covered entirely by the patient. Anti-HPV drug treatment costs 1000 to 20,000 yuan, fully out-of-pocket. Cervical surgery requires a one-day hospital stay, takes under 30 min under general anesthesia, and costs about 5000 yuan, with 40% covered by insurance.

Side effects in the PDT group were milder. 85% of patients reported increased vaginal discharge, 12.0% had mild vaginal bleeding, and 38.0% had mild abdominal pain. All symptoms resolved within 3–7 days and were well tolerated. No cases of skin photosensitivity or hyperpigmentation were observed during or after PDT. Figure 2 presents colposcopic images of the cervix before and after PDT. It is evident that the cervical appearance improves significantly post-treatment, with no scarring observed. Ten patients conceived successfully after PDT treatment, with no cervical-related pregnancy terminations. 5 of them have delivered without complications.

### 3.5. Logistic Regression Analysis of Factors for Lesion Remission and HPV Clearance

The association between baseline clinical characteristics and therapeutic efficacy was analyzed between two groups. Based on the univariate analysis results (Table 4), variables demonstrating statistical significance were incorporated into a multivariate regression model. The analysis revealed that lesion regression in the PDT group was significantly associated with age, immune-related diseases, and lower genital tract infections, whereas HPV clearance was significantly linked to active smoking. In the observation group, multiple HPV infections emerged as a significant predictor of suboptimal lesion regression and HPV clearance (*p* < 0.001, Table 5).

## 4. Discussion

PDT uses light, a photosensitizer, and oxygen to selectively destroy abnormal tissue through photodynamic reactions, achieving targeted therapy. 5-ALA, a second-generation photosensitizer, is absorbed mainly by rapidly dividing cervical cells and converted into PpIX, a natural photosensitive compound. When exposed to light of a specific wavelength, PpIX generates reactive oxygen species (ROS), such as singlet oxygen and free radicals. These ROS cause oxidative damage to cell membranes, mitochondria, and DNA, leading to the death of precancerous cells while sparing healthy tissue^6^. Beyond direct cytotoxicity, PDT simultaneously modulates the cervical microenvironment to facilitate HPV clearance. It remodels local immune suppression by activating immune cells and inflammatory cytokines, and optimizes vaginal flora by restoring lactobacillus abundance to inhibit viral persistent colonization [10]. Moreover, PpIX is cleared from the body within 24 h, reducing photosensitivity and allowing patients to return to normal activities quickly. Originally used in dermatology for conditions like condyloma acuminatum and Bowenoid papulosis, PDT has become a promising treatment for HPV-related cervical lesions, showing strong clinical results [11].

Given these favorable properties, the selection of an appropriate 5-ALA concentration and delivery formulation is critical for optimizing treatment outcomes. Early PDT studies using lower 5-ALA concentrations (5–10%) showed suboptimal efficacy, whereas 20% 5-ALA has been established as effective in multiple prospective and randomized studies [12,13]. A head-to-head comparison of 10% vs. 20% ALA-PDT for cervical lesions confirmed superior HPV clearance with the higher concentration. The 10–20% range is generally safe, and 20% 5-ALA reaches peak cervical tissue absorption; higher concentrations or longer application times do not further increase tissue accumulation. The 19% formulation used in our center reflects the standard reconstitution of commercially available ALA powder (354 mg in 1.5 mL thermogel) and falls within this well-established 10–20% range. To ensure stable and prolonged contact with the cervical mucosa, a poloxamer 407 (Pluronic F127)-based thermosensitive hydrogel was used as the delivery vehicle [14]. Poloxamer 407 is a PEO–PPO–PEO triblock copolymer that remains liquid at 2–8 °C and undergoes a sol–gel transition upon contact with body temperature surfaces, ensuring easy application and prolonged residence on the cervical mucosa [14,15]. 1,3-Propanediol and 1,2-hexanediol serve as humectants and co-solvents [16]. The density of poloxamer 407-based thermogels is approximately 1.06 g/cm^3^ at 25 °C, close to that of water [14]. These properties help maintain stable contact between the photosensitizer and cervical tissue during the incubation period.

As cervical cancer screening becomes more widespread and strategies improve, more young women with cervical LSILs are being diagnosed. While current guidelines recommend observation rather than immediate treatment, LSIL can progress to high-grade lesions. The risk of persistence and progression increases with age, with over 50% of LSIL cases in women over 30 not regressing within two years [17]. Most patients choose observation due to concerns about overtreatment and potential harm to cervical function. Currently, the primary treatment options for LSILs are LEEP and cervical conization. However, our prior research has demonstrated that cervical scar formation occurs in 20% of patients following LEEP and in as high as 83.9% following conization [18,19]. These structural alterations may compromise cervical integrity, impair fertility, and increase the risk of miscarriage and preterm birth. However, a treatment that effectively addresses LSILs with Hr-HPV infection while preserving cervical integrity would be valuable [20].

Since 2019, our center has employed ALA-PDT for the treatment of patients with cervical precancerous lesions and HPV infection. A retrospective analysis of cases with complete follow-up data revealed that ALA-PDT achieved a lesion regression rate of 92.5%, significantly higher than that of the untreated observation group (48.3%), representing a 44% improvement (92.5% vs. 48.3%). A study conducted at the International Peace Maternity and Child Health Hospital reported an effective rate of 76.39% for PDT in treating LSILs, compared to 28.57% in the observation group [21]. Among the limited number of prospective studies, Chen Y et al. observed a lesion response rate of 75.46% six months after ALA-PDT, in contrast to only 21.36% in the control group [22].

The high PDT success rate in our center is likely due to three factors: (1) all patients had transformation zone type I or II with visible lesions under colposcopy; (2) the photosensitizer and light were applied to both the cervical surface and canal simultaneously; and (3) continuous colposcopic monitoring ensured accurate lesion targeting, precise drug delivery, and effective illumination [23,24]. Regarding the second factor, although patients with visible endocervical lesions were excluded, the cervical canal frequently harbors occult HPV infection, which may serve as a reservoir for persistence or recurrence. Combined ectocervical and endocervical photosensitizer application followed by dual irradiation has been shown in prior studies and in our clinical experience to improve HPV clearance rates. The cylindrical diffuser, approximately the diameter of a urethral catheter, enables uniform circumferential light delivery with minimal discomfort. This capacity to comprehensively treat both sites represents a key advantage of ALA-PDT and likely contributed to the high clearance rate observed.

Hr-HPV infection is the main cause of cervical LSILs. Clearing HPV can resolve cervical lesions and prevent recurrence. PDT works by using ALA to trigger cell death and boost local immunity, which may help eliminate HPV. A study at Peking University Third Hospital found a 72.5% HPV clearance rate in LSIL patients after 6 months of PDT [25]. Another study at Renji Hospital reported an 82.54% HPV-negative conversion rate 6 months post-treatment [9]. Our results align with these findings: among 608 treated patients, 80.8% had HPV clearance, compared to only 37.3% in the untreated control group (Figure 1).

After HPV infection, the viral DNA initially exists freely in the host cell cytoplasm. During cell division, HPV DNA may integrate into the human genome—a process called HPV integration. This integration is a key step in cervical cell immortalization and the progression from cervical intraepithelial neoplasia to cancer [26]. Research indicates that HPV integration can predict cancer progression and recurrence after treatment, suggesting its potential as a screening marker for photodynamic therapy [27]. It can help identify patients who may not benefit from PDT, allowing for timely alternative treatments while preserving fertility [28].

In clinical practice, HPV clearance remains difficult even after effective treatment of precancerous lesions with cervical conization [21]. HPV results can remain positive after hysterectomy. This may be due to factors such as immune status, the vaginal microenvironment, and other clinical conditions. Our study identified several factors linked to persistent HPV infection: immune-related diseases (e.g., systemic lupus erythematosus, Sjogren’s syndrome), concurrent infections like vaginitis or cervicitis (e.g., mycoplasma or chlamydia), and unhealthy lifestyle behaviors (e.g., smoking, multiple sexual partners). These factors may hinder HPV clearance. However, the present study did not systematically characterize the cervical microenvironment; thus, the precise mechanisms by which local immunity and the vaginal microbiota influence HPV clearance after PDT remain to be elucidated. To address this, future prospective studies should incorporate standardized collection of cervical microenvironment markers before and after PDT, including local immune cell profiles (e.g., CD4+ and CD8+ T cells, regulatory T cells), key cytokines (e.g., IFN-γ, IL-2, IL-10, TGF-β), and vaginal microbiome composition assessed by 16S rRNA sequencing or metagenomics. Correlating these biomarkers with treatment outcomes will help identify predictive signatures and refine patient selection criteria, thereby strengthening the evidence base for PDT application. Our multivariate regression analysis showed that the HPV clearance effect in the PDT group was significantly associated with active smoking, while multiple HPV infections were an independent influencing factor for poor lesion outcome in the observation group.

Although our study demonstrates favorable 6-month efficacy of PDT for LSILs with HR-HPV infection, the high spontaneous regression rate of LSIL necessitates caution in interpreting these short-term results. A long-term study by Inada et al. demonstrated sustained efficacy of PDT for CIN treatment over extended follow-up, supporting its durable therapeutic effect [29]. A systematic review and meta-analysis by Zhang et al. further confirmed the efficacy and safety of PDT for cervical intraepithelial neoplasia and HPV infection across multiple randomized clinical trials, with pooled analysis showing robust HPV clearance and lesion regression at 6, 12, and 24 months [30]. These data suggest that the treatment benefit observed in our study may be sustained over time. However, we acknowledge that the absence of follow-up beyond 6 months is a key limitation of the present study, as it precludes direct assessment of long-term durability and differentiation between treatment effect and late spontaneous regression. Future long-term prospective studies with standardized follow-up at 12 and 24 months are warranted to confirm sustained lesion regression and viral clearance rates.

PDT causes only mild, temporary side effects. As shown in the Figure 2, cervical morphology remains unchanged after treatment, consistent with earlier findings. It does not lead to pregnancy complications such as cervical insufficiency [31]. In this study, 10 patients became pregnant after PDT, with no cervical-related pregnancy losses; 5 have already given birth. However, PDT has higher costs and time demands. For patients who can afford it and have sufficient time, 5-ALA-PDT is a preferred non-invasive treatment for LSILs caused by Hr-HPV.

To enhance clinical translational value, we further summarized the indications and precautions for ALA-PDT. This therapy is primarily applicable to fertility-preserving women with persistent HR-HPV infection and LSILs. Preoperative colposcopy combined with endocervical curettage is mandatory to exclude occult endocervical lesions. Additionally, pre-treatment intervention and health education are recommended for patients with immune disorders, genital tract infections, or unhealthy behavioral risk factors. Given its high cost and repeated treatment cycles, PDT is not prioritized for patients with poor economic affordability or low compliance. These applicable criteria provide concise and practical clinical guidance for individualized LSIL management [32].

This study has several limitations, including its retrospective design, single-center setting, and small sample size. Furthermore, the current follow-up duration is limited to six months, which may be insufficient to capture long-term outcomes. Ongoing follow-up is being conducted, with the goal of analyzing two-year endpoints at the earliest opportunity. To address the limitations of retrospective analyses, a multicenter prospective study is currently in preparation.

## 5. Conclusions

Our study shows that ALA-PDT significantly improves lesion regression and HPV clearance in patients with cervical LSILs compared to observation. The therapy is highly selective, with clear efficacy, minimal side effects, and preservation of cervical and vaginal structure and function. It is especially suitable for women of reproductive age. Nevertheless, it remains crucial to identify the specific patients who are most likely to derive clinical benefit from ALA-PDT.

## Figures and Tables

**Figure 1:**
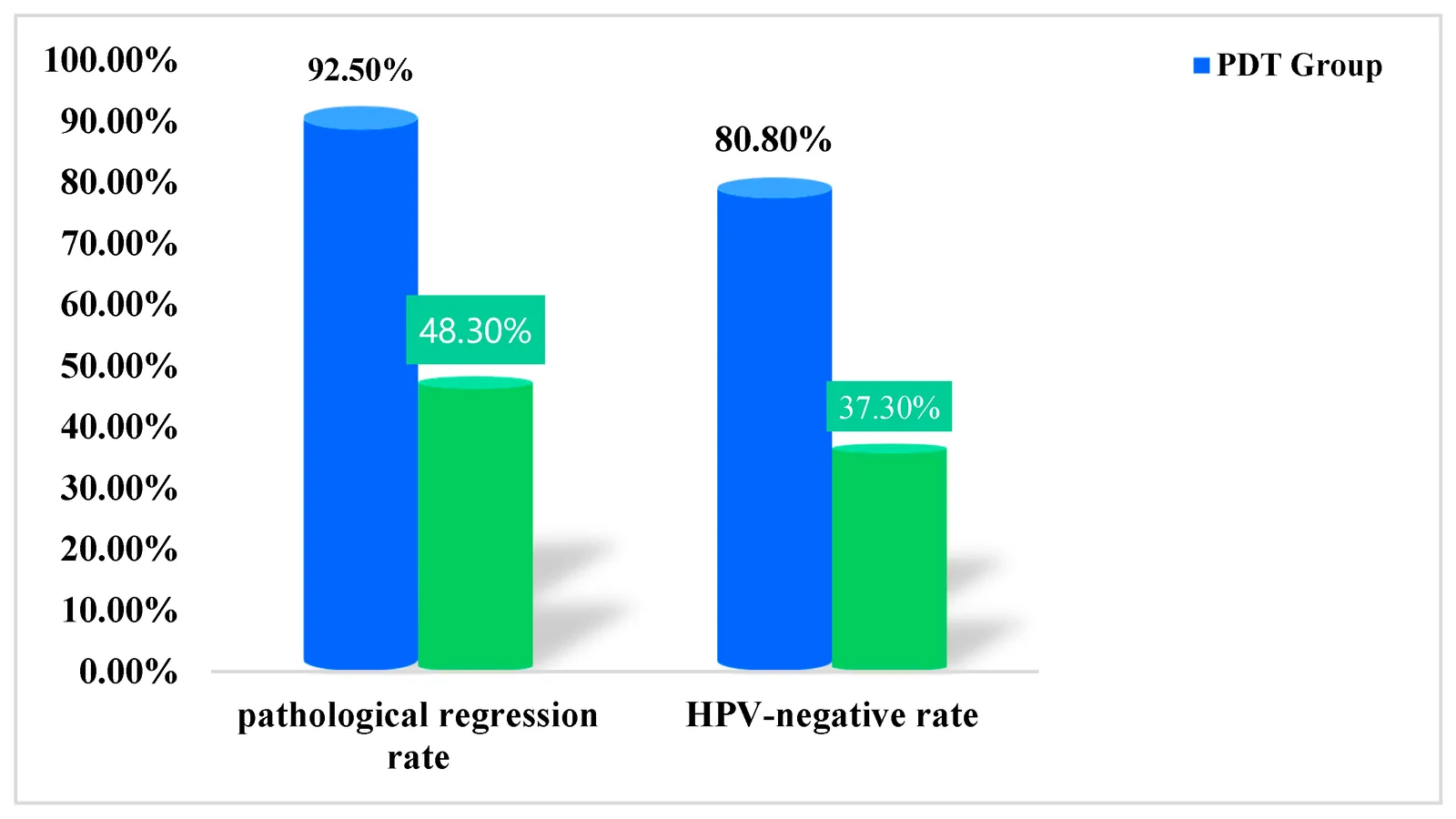
Comparison of the efficacy between two groups at 6 months follow-up.

**Figure 2:**
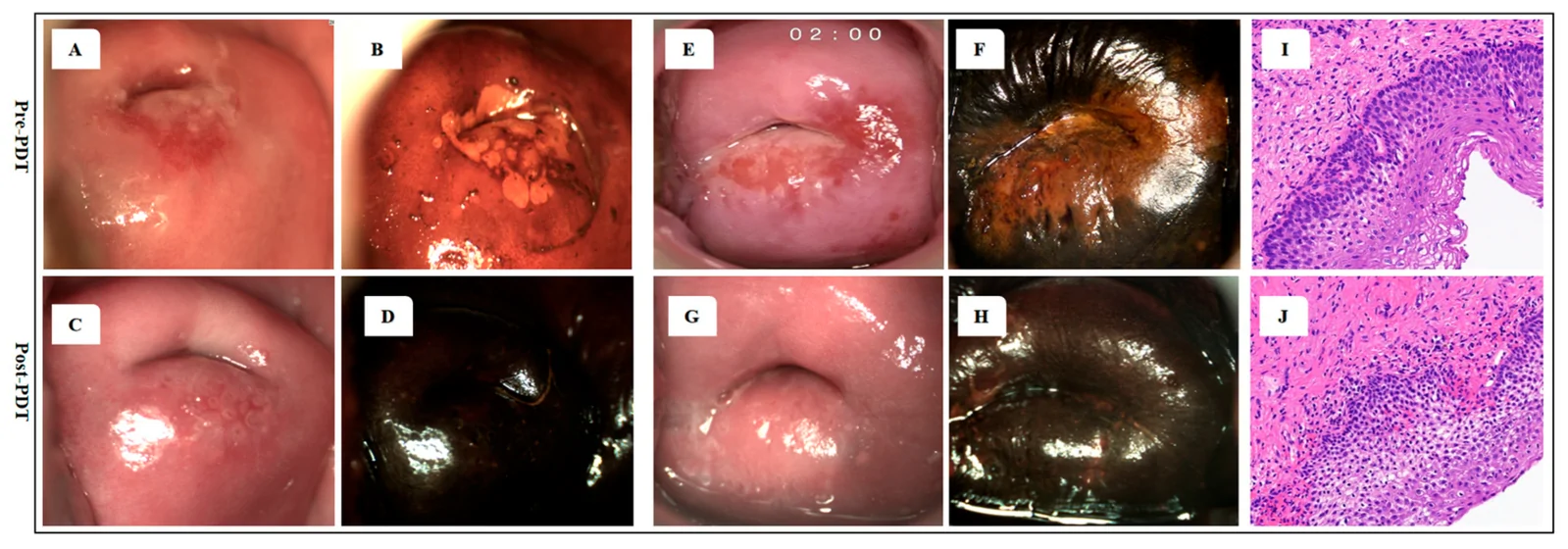
Colposcopic Images of cervix (Before/After PDT). (**A**–**D**): 23-year-old patient with HPV16/18-positive and LSIL; (**A**,**B**) colposcopic images of LSIL before PDT treatment (×15); (**C**,**D**) colposcopic images after PDT treatment (×15), with re-epithelialization of cervical ectropion; (**E**–**H**): 28-year-old patient with HPV18-positive status and LSIL; (**E**,**F**) colposcopic images of LSIL before PDT treatment (×15); (**G**,**H**) colposcopic images after PDT treatment (×15), with iodine re-coloration of cervical ectropion. (**I**,**J**): Histological images of cervix (H&E staining, ×200); (**I**), Representative histopathological image of LSIL. The squamous epithelium shows koilocytosis with perinuclear halos, nuclear enlargement, and mild dysplasia confined to the lower one-third of the epithelium. (**J**), Representative histopathological image of the cervix 6 months after ALA-PDT. The squamous epithelium shows normal maturation and stratification, with regular basal cell polarity, absence of koilocytosis, and no evidence of dysplasia.

**Table 1:** Clinical Characteristics of the Study Population.

	PDT Group (N = 308)	Observation Group (N = 300)	*p*
Age (years)			0.36
Mean ± SD	35.64 ± 10.50	36.65 ± 10.94	
Range	20–68	21–62	
Age group, n (%)			0.52
20–29	103	102	
30–39	111	95	
≥40	94	103	
Smoking, n (%)			0.58
Yes	14	10	
No	294	290	
immune-related diseases, n (%)			0.81
Yes	7	5	
No	301	295	
Lower genital tract infection, n (%)			0.12
Yes	20	31	
No	288	269	
Parity, n (%)			0.34
Yes	136	120	
No	172	180	
Multiple sexual partners *, n (%)			0.95
Yes	9	10	
No	299	290	
HPV type, n (%)			0.39
HPV 16/18	141	126	
Non-16/18	167	174	
HPV infection pattern, n (%)			0.54
Single	133	138	
Multiple	175	162	
TCT, n (%)			0.14
NILM	178	192	
ASC-US/LSIL	130	108	

* Multiple sexual partners was defined as having two or more sexual partners in the past year. *p* values were calculated using chi-square tests for categorical variables. For age, the *p* value refers to the comparison of age-group distribution (20–29, 30–39, and ≥40 years) between the two groups. Mean ± SD and range are presented as descriptive statistics.

**Table 2:** Comparison of the Lesion Remission Between Two Groups.

		PDT Group (N = 308)	Observation Group (N = 300)	*p*
Effective	Cure	285 (92.5%, 89.0–95.2%)	145 (48.3%, 42.6–54.1%)	<0.00 *
Ineffective		23 (7.5%, 4.8–11.0%)	155 (51.7%, 45.9–57.4%)	
	Persistence	21 (6.8%, 4.3–10.2%)	120 (40.0%, 34.4–45.8%)	
	Progression	2 (0.65%, 0.08–2.3%)	35 (11.7%, 8.3–15.9%)	

*, *p* < 0.05.

**Table 3:** Comparison of the HPV-negative rates between two groups.

		PDT Group (N = 308)	Observation Group (N = 300)	*p*
Negative		249 (80.8%, 76.0–85.1%)	112 (37.3%, 31.8–43.1%)	<0.00 *
Positive		59 (19.2%, 14.9–24%)	188 (62.7%, 56.9–68.2%)	
	Partial negative	20 (6.5%, 4.0–9.9%)	56 (18.7%, 14.4–23.5%)	
	Persistence	39 (12.7%, 9.2–16.9%)	132 (44.0%, 38.3–49.8%)	

*, *p* < 0.05.

**Table 4:** Univariate analysis of risk factors for lesion remission and HPV clearance.

Clinical Characteristics	PDT Group				Observation Group			
	Lesion Remission		HPV Clearance		Lesion Remission		HPV Clearance	
	OR (95%CI)	*p*	OR (95%CI)	*p*	OR (95%CI)	*p*	OR (95%CI)	*p*
Age	0.96 (0.92–0.99)	0.03 *	1.00 (0.98–1.03)	0.90	0.81 (0.77–0.84)	1.80 × 10^−18^ *	0.72 (0.66–0.77)	4.31 × 10^−17^ *
Smoking	0.12 (0.04–0.41)	0.00 *	0.01 (0.00–0.07)	5.19 × 10^−5^ *	4.47 (1.10–29.94)	0.06	1.02 × 10^−7^	0.98
Immune-related diseases	0.03 (0.00–0.12)	2.51 × 10^−5^ *	1.33 × 10^−8^	0.98	1.80 × 10^−7^	0.98	2.84 × 10^−7^	0.98
Parity	1.60 (0.28–3.06)	0.65	1.90 (0.62–8.32)	0.32	0.08 (0.01–0.32)	0.01 *	0.06 (0.01–0.30)	0.01 *
Sexual partners	0.08 (0.02–0.37)	0.00 *	0.06 (0.01–0.25)	0.00 *	0.11 (0.01–0.61)	0.04 *	1.02 × 10^−7^	0.98
Lower genital tract infection	0.05 (0.02–0.13)	8.32 × 10^−9^ *	3.68 × 10^−9^	0.98	0.03 (0.01–0.14)	0.00 *	3.29 × 10^−8^	0.98
HPV16/18	1.34 (0.56–3.31)	0.51	1.09 (0.62–1.94)	0.77	18.35 (10.30–34.06)	1.10 × 10^−21^ *	25.46 (13.84–49.15)	9.66 × 10^−24^ *
Single HPV	0.46 (0.18–1.08)	0.08	1.04 (0.59–1.86)	0.89	59.86 (59.67–72.74)	7.82 × 10^−25^ *	199.21 (69.02–847.95)	1.20 × 10^−17^ *
TCT	0.71 (0.28–1.69)	0.46	0.92 (0.51–1.64)	0.79	0.35 (0.21–0.56)	2.41 × 10^−5^ *	0.32 (0.19–0.52)	4.97 × 10^−6^ *

*, *p* < 0.05.

**Table 5:** Logistic regression analysis of factors for lesion remission and HPV clearance.

Clinical Characteristics	PDT Group				Observation Group			
	Lesion Remission		HPV Clearance		Lesion Remission		HPV Clearance	
	OR (95%CI)	*p*	OR (95%CI)	*p*	OR (95%CI)	*p*	OR (95%CI)	*p*
Age	0.92 (0.88–0.96)	0.00 *			0.97 (0.80–1.15)	0.73	0.97 (0.80–1.15)	0.72
Smoking	0.68 (0.09–6.41)	0.72	0.02 (0.00–0.11)	0.00 *	—	—	—	—
Immune-related diseases	0.06 (0.00–0.43)	0.01 *	—	—	—	—	—	—
Parity	—	—	—	—	0.24 (0.05–5.16)	0.39	0.24 (0.01–5.16)	0.39
Sexual partners	0.15 (0.02–1.12)	0.05	0.16 (0.02–1.15)	0.06	—	—	—	—
Lower genital tract infection	0.07 (0.02–0.32)	0.00 *	—	—	—	—	—	—
HPV16/18	—	—	—	—	2.13 (1.51–7.82)	0.20	3.14 (0.52–8.12)	0.20
Single HPV	—	—	—	—	23.05 (2.31—55.79)	<0.00 *	12.30 (4.17–64.55)	<0.00 *

*, *p* < 0.05.

## Data Availability

The data that support the findings of this study are available on request from the corresponding authors. The data are not publicly available due to privacy or ethical restrictions.

## Abbreviations

The following abbreviations are used in this manuscript:

HPV	human papillomavirus
CIN	cervical intraepithelial neoplasia
PDT	photodynamic therapy
LSILs	low-grade squamous intraepithelial lesions
